# Psoriatic arthritis: exploring the occurrence of sleep disturbances, fatigue, and depression and their correlates

**DOI:** 10.1186/s13075-020-02294-w

**Published:** 2020-08-26

**Authors:** Glenn Haugeberg, Mari Hoff, Arthur Kavanaugh, Brigitte Michelsen

**Affiliations:** 1 Division of Rheumatology, Department of Medicine, Sorlandet Hospital, P.O.Box 416, N-4604 Kristiansand S, Norway; 2 Department of Neuroscience, Division of Rheumatology, Norwegian University of Science and Technology, Trondheim, Norway; 3 Department of Medicine, School of Medicine, University of California, San Diego, La Jolla, CA USA

**Keywords:** Psoriatic arthritis, Sleep disturbance, Fatigue, Depression

## Abstract

**Introduction:**

Sleep disturbances, fatigue, and anxiety/depression in psoriatic arthritis (PsA) may be influenced by skin and musculoskeletal manifestations. All of these in turn affect the psychosocial impact of disease. The objective was to explore the occurrence of sleep disturbances, fatigue, and anxiety/depression in psoriatic arthritis (PsA) patients, and their correlates.

**Methods:**

A broad data collection was performed in 137 Norwegian PsA outpatient clinic patients including demographics, disease activity measures for both skin and musculoskeletal involvement, and patient-reported outcome measures. Sleep disturbances and fatigue were defined present if the numeric rating scale (0–10) score was ≥ 5. Anxiety/depression was assessed using a questionnaire (1–3; 1 defined as no anxiety/depression). Descriptive statistics was applied, and associations were explored using univariate and adjusted linear regression analysis.

**Results:**

The mean age was 52.3 years, PsA disease duration 8.8 years; 49.6% were men and 54.8% were currently employed/working. The prevalence of sleep disturbances was 38.0%, fatigue 44.5%, and anxiety/depression 38.0%. In adjusted analysis, pain, fatigue, and higher mHAQ were associated with sleep disturbances. Sleep disturbances, pain, and anxiety/depression were associated with fatigue, whereas only fatigue was associated with anxiety/depression.

**Conclusions:**

The prevalence of sleep disturbances, fatigue, and anxiety/depression was frequently reported by PsA patients. No measures reflecting skin involvement or objective measures of musculoskeletal involvement were independently associated with sleep disturbances, fatigue, or anxiety/depression. Our data suggest that patients’ perceptions of musculoskeletal involvement (pain or mHAQ) play an important role causing sleep disturbances and fatigue, whereas fatigue in PsA patients is strongly associated with anxiety/depression.

## Introduction

The clinical presentation in psoriatic arthritis (PsA) is heterogeneous involving the musculoskeletal system, nails and skin, and other domains [1]. Health-related quality of life (HRQoL) has been shown to be impaired among patients with both psoriasis (PsO) [2] and PsA [3]; however, impairment has been shown to be greater in PsA [4, 5]. For PsA patients to reach optimal improvement in HRQoL, improvement in both skin and musculoskeletal involvement must be achieved [6]. The increased psychosocial burden in PsA also contributes to impaired HRQoL [7] as may comorbidities [8]. PsA patients are thought to suffer more from sleep disturbances, fatigue, anxiety, and depression, which may also have a negative influence on HRQoL [7]. From the patient perspective, PsA patients rate sleep disturbances, fatigue, depression, and anxiety to be of greater importance than do doctors [9, 10]. Despite their importance, there is a paucity of relevant data and thus a need to better understand the interaction and relationship between various physical and psychosocial features and their impact also on sleep, fatigue, anxiety, and depression in PsA patients.

The aim of this cross-sectional study was to illuminate the prevalence of sleep disturbances, fatigue, and anxiety/depression in PsA patients in an outpatient clinic and to explore associations with demographic characteristics, and activity measures reflecting skin, musculoskeletal involvement, and comorbidities.

## Methods

A total of 141 PsA patients, all fulfilling the Classification of Psoriatic Arthritis criteria (CASPAR), were consecutively recruited from a Norwegian outpatient clinic. We have previously described study design and data collection in detail [11]. In this study, we explore data from 137 of the 141 PsA patients who had data available for sleep disturbances, fatigue, and depression, the dependent variables in the present analysis.

On a numeric rating scale (NRS), patients reported their sleep difficulties (i.e., resting at night) during the last week (0 no difficulty to 10 extreme difficulty). Fatigue during the last week was also reported on a NRS (0 no fatigue to 10 totally exhausted). For the assessment of anxiousness and depression, we used question 5 in the EQ-5D-3L with three response alternatives: I am not (score 1), I am moderately (score 2), and I am extremely anxious or depressed (score 3) [12].

In Table 1, data is shown for the dependent and the independent variables. In short, data collection for the independent variables included demographic variables (age, gender, body mass index (BMI), smoking, living together, employed/working status), disease duration, C-reactive protein (CRP), clinical measures reflecting musculoskeletal inflammation (68 tender joint count (TJC68), 66 swollen joint count (SJC66), Maastricht Ankylosing Spondylitis Enthesitis Score (MASES)), pain reported on a visual analogue scale 0–100 mm, physical function assessed by the modified Health Assessment Questionnaire (mHAQ), exercise, and comorbidities. For exercise, the patients were categorized into (1) those who performed exercise 1–2 times per week or more and (2) those who did less exercise than 1–2 times per week or did not exercise at all. A summed score for comorbidities was calculated based on the presence of cardiovascular disease (CVD), pulmonary disease, neurological disease, urogenital disease, gastrointestinal disorders, endocrine disorders, cancer, and mental disorder (range 0–8).

**Table 1:** Baseline characteristics of all psoriasis arthritis patients and for men and women separately. The number of patients in the analyses is 137 if not otherwise indicated

	Total (*n* = 137)	Men (*n* = 68) 49.6%	Women (*n* = 69) 50.4%	*p* value
Demographics				
Age, years, mean (SD)	52.3 (10.3)	51.8 (10.4)	52.8 (10.4)	0.56
BMI, kg/m^2^ (*n* = 135), mean (SD)	28.4 (4.3)	28.5 (3.9)	28.2 (4.8)	0.67
Currently smoking, number (%)	24 (17.5%)	9 (13.2%)	15 (21.7%)	0.19
Living together (*n* = 134), number (%)	105 (76.6%)	55 (82.1%)	50 (74.6%)	0.29
Currently employed/working* (*n* = 135), number (%)	74 (54.8%)	43 (64.2%)	31 (45.6%)	0.030
Musculoskeletal disease measures				
PsA disease duration, years, mean (SD)	8.8 (6.8)	8.6 (7.3)	9.0 (6.3)	0.70
CRP, mg/L, mean (SD)	5.0 (8.3)	6.6 (10.7)	3.4 (4.4)	0.024
TJC68, mean (SD)	10.1 (11.1)	7.8 (10.1)	12.3 (11.6)	0.023
SJC66, mean (SD)	0.6 (1.0)	0.6 (1.2)	0.6 (0.9)	0.91
MASES, range 0–13, mean (SD)	3.0 (3.2)	1.9 (2.5)	4.0 (3.4)	< 0.001
PROs				
Pain, VAS 0–100 mm, mean (SD)	34.8 (23.3)	30.2 (21.9)	39.4 (24.0)	0.021
Fatigue, NRS 0–10, mean (SD)	4.2 (2.6)	3.6 (2.5)	4.8 (2.6)	0.009
Sleep disturbance^†^, NRS 0–10, mean (SD)	3.5 (2.9)	2.8 (2.7)	4.1 (3.0)	0.010
Depression, range 1–3, mean (SD)	1.4 (0.4)	1.3 (0.5)	1.5 (0.6)	0.069
MHAQ, range 0–3, mean (SD)	0.44 (0.40)	0.36 (0.33)	0.51 (0.44)	0.025
Exercise ≥ 1 time per week, number (%)	62 (45.3%)	30 (44.1%)	32 (46.4%)	0.79
Comorbidity				
Comorbidities (*n* = 136) (range 0–8) mean (SD)	0.72 (0.93)	0.51 (0.76)	0.91 (1.04)	0.012
Skin				
PASI, range 0–72 (*n* = 136), mean (SD)	2.6 (3.7)	3.3 (4.2)	1.9 (3.0)	0.030
Skin itching, number (%) (*n* = 121)††	28 (23.1%)	16 (26.2%)	12 (20.0%)	0.417
Treatment				
Current bDMARD, number (%) (*n* = 136)	44 (32.4%)	27 (39.7%)	17 (25.0%)	0.067
Current csDMARD, number (%)	80 (58.4%)	43 (63.2%)	37 (53.6%)	0.25

Continuous variables are expressed as mean (standard variation); categorical variables are expressed as numbers (proportions). In the group comparisons, the independent sample *t* test was used for continuous variables and the chi-square test for categorical variables

*BMI* body mass index, *PsA* psoriatic arthritis, *ESR* erythrocyte sedimentation rate, *CRP* C-reactive protein, *TJC* tender joint count, *SJC* swollen joint count, *DAPSA* Disease Activity index for Psoriatic Arthritis, *PASDAS* Psoriatic Arthritis Disease Activity Score, *MASES* Maastricht Ankylosing Spondylitis Enthesitis Score, *IGA* Investigator Global Assessment, *VAS* visual analogue scale, *PROs* patient-reported outcome measures, *PGA* patient global assessment, *NRS* numeric rating scale, *MHAQ* Modified Health Assessment Questionnaire, *PASI* Psoriasis Area Severity Index, *DLQI* Dermatology Life Quality Index, *bDMARDs* biologic disease-modifying anti-rheumatic drugs, *csDMARDs* conventional synthetic DMARDs

*Not employed/working: part time/full time sick leave, disabled pensioner or pensioner

^†^The sleep question is phrased as follows: “Select the number that best describes the sleep difficulties (i.e. resting at night) you felt due to your arthritis during the last week (from 0 (no difficulty) to 10 (extreme difficulty))”

^††^Skin itching: no itching (25 patients) and little itching (68 patients) were grouped as no itching and a lot itching (22 patients) and very much itching (6 patients) as itching

PsO skin involvement was assessed by the Psoriasis Area Severity Index (PASI), skin itching (see below), and current treatment with disease-modifying anti-rheumatic drugs (DMARDs) both conventional synthetic DMARDs (csDMARDs) and biologic DMARDs (bDMARDs).

We used the first question in the Dermatology Life Quality Index to explore for skin itching. This question is phrased: “Over the last week, how itchy, sore, painful or stinging has your skin been? with possible answers: Not at all (score 0), a little (score 1), a lot (score 2) and very much (score 3).” For analysis, the variable was dichotomized. Scores 0 and 1 were defined as not itching and 2 and 3 as itching.

In the present study, we wanted to explore the single elements that may impact sleep, fatigue, and depression in PsA. Thus, we excluded HRQoL measures, composite disease activity scores, and global scores both investigator and patient global assessment which previously has been reported [11, 13].

### Statistical analysis

The Statistical Package for Social Science (SPSS) for Windows was used for statistical analyses. Continuous variables are presented as mean with standard deviation (SD) and categorical variables in numbers and percentages (%). Comparisons between two groups were analyzed using the chi-squared test for categorical variables and independent samples *t* test for continuous variables.

We also calculated median with interquartile range for the NRS for sleep disturbances and fatigue and calculated percentage of patients with a NRS score ≥ 5 both for sleep disturbances and fatigue. For the anxiety/depression question, the percentage of PsA patients answering the three alternatives was also calculated.

To explore for inter-correlation between the variables, we used the Pearson correlation coefficient (*r*). For variables reflecting the same domain, e.g., pain, MASES score, and TJC68, we also calculated *Z* scores by dividing the values with the SD of the mean to facilitate in-between comparability for these independent variables.

Associates with sleep, fatigue, and anxiety/depression as dependent variables were explored in univariate and multivariate linear regression analyses. To be included in the multivariate model, the *p* value in the univariate analysis for the tested variables listed in Table 1 had to be < 0.20. Independent of the univariate results, we adjusted for age, gender, and BMI in the model. For sleep disturbances, the adjusted model was also tested without fatigue in the model. Further, in the model for anxiety/depression, we tested for interaction between pain, sleep disturbances, and fatigue. For robustness, we also tested the multivariate models with forward procedure.

The level of significance was set at *p* < 0.05.

## Results

In Table 1, the characteristics for the PsA patients are shown for all 137 patients and for the 68 males and 69 females separately. Mean values for sleep disturbances were 3.5 (men 2.8 vs women 4.1, *p* = 0.01), for fatigue 4.2 (men 3.6 vs 4.8, *p* = 0.01), and for anxiety/depression 1.4 (men 1.3 vs women 1.5, *p* = 0.07). The mean age was 52.3 years, mean BMI 28.4 kg/m^2^, 49.6% were men, 17.5% were smokers, 76.6% were self-described as living together, 54.8% were currently employed/working, and disease duration was 8.8 years. For these variables, a significant gender difference was only found for currently working (men 64.2% vs women 45.6%, *p* = 0.03). As shown in Table 1, no significant gender differences were found for SJC66, skin itching, anxiety/depression, exercise, and treatment. In men compared with women, worse scores were found for CRP and PASI score, and worse scores were seen in women for TJC68, MASES score, pain, fatigue, sleep disturbances, MHAQ, and comorbidities.

Median [IQR] values for NRS for sleep disturbance were 3 [5] and for fatigue 4 [4]. The number and percentage (%) of PsA patients with a NRS ≥ 5 was for sleep disturbances 52 (38.0%) and for fatigue 61 (44.5%). The numbers and percentage (%) of patients reporting not to be depressed were 85 (62.0%), moderately anxious or depressed 45 (32.8%), and extremely anxious or depressed only 7 (5.1%).

The Pearson correlation coefficient between the dependent variables was all highly significant (*p* < 0.001) and was between sleep disturbances and fatigue 0.632, between fatigue and anxiety/depression 0.449, and between sleep disturbances and anxiety/depression 0.308. For other variables listed in Table 1, with at least a significant moderate strong correlation (> 0.5) was found for TJC66 and MASES score (0.549), pain and MHAQ (0.650), pain and fatigue (0.629), pain and sleep disturbances (0.622), MHAQ and sleep disturbances (0.564), and MHAQ and fatigue (0.504). The strongest association with anxiety/depression was found for fatigue (0.449). No strong correlation was found between any of the dependent and independent variables (correlation coefficient > 0.7).

In univariate analysis as shown in Table 2, a statistically significant association with sleep disturbance was found with female gender, higher BMI, smoking, not currently working, TJC68, MASES score, pain, fatigue, anxiety/depression, higher MHAQ, and less exercising. For more fatigue, an association was found with female gender, not being employed/working, TJC68, MASES score, pain, sleep disturbances, anxiety/depression, and MHAQ, and with more anxiety/depression, an association was found with smoking, not currently working, pain, fatigue, sleep disturbances, and MHAQ. In the univariate linear regression analysis with sleep disturbances as a dependent variable using *Z* scores for the independent variables, the *B* (95% CI) values were 1.82 (1.43, 2.21) for pain, 0.94 (0.47, 1.40) for MASES score, and 0.82 (0.34, 1.30) for TJC68 and for fatigue as the dependent variable 1.65 (1.30, 1.99), 0.92 (0.51, 1.33), and 0.65 (0.22, 1.08), respectively. Higher *B* values for *Z* score variables mean stronger association with the dependent variable with highest scores for pain.

**Table 2:** Associations with sleep disturbance, fatigue, and depression in psoriatic arthritis patients tested in univariate and multivariate linear regression models

	Sleep disturbance NRS (0–10)				Fatigue NRS (0–10)				Depression (0–3)			
	Univariate	*p*	Adjusted	*p*	Univariate		Adjusted	*p*	Univariate		Adjusted	*p*
	*B* (95% CI)		*B* (95% CI)		*B* (95% CI)		*B* (95% CI)		*B* (95% CI)		*B* (95% CI)	
Demographics												
Age, years, mean (SD)	− 0.003 (− 0.051, 0.045)	0.905	− 0.013 (− 0.052, 0.027)	0.525	− 0.032 (− 0.074, 0.011)	0.148	− 0.021 (− 0.055, 0.013)	0.227	− 0.001 (− 0.011, 0.009)	0.854	0.001 (− 0.009, 0.011)	0.911
Female	1.278 (0.311, 2.245)	0.010	0.387 (− 0.462, 1.237)	0.368	1.165 (0.297, 2.033)	0.009	0.115 (− 0.622, 0.852)	0.758	0.184 (− 0.015, 0.382)	0.069	0.041 (− 0.161, 0.243)	0.689
BMI, kg/m^2^ (*n* = 135), mean (SD)	0.159 (0.047, 0.270)	0.006	0.079 (− 0.019, 0.177)	0.113	0.102 (− 0.001, 0.204)	0.052	− 0.020 (− 0.106, 0.067)	0.656	0.009 (− 0.014, 0.032)	0.447	0.001 (− 0.023, 0.025)	0.944
Currently smoking, number (%)	1.909 (0.646, 3.172)	0.003	− 0.154 (− 1.278, 0.970)	0.786	1.538 (0.397, 2.679)	0.009	0.238 (− 0.716, 1.193)	0.621	0.337 (0.079, 0.594)	0.011	0.100 (− 0.167, 0.366)	0.461
Living together (*n* = 134), number (%)	0.386 (− 0.828, 1.600)	0.531	–	–	0.219 (− 0.884, 1.322)	0.695	–	–	− 0.196 (− 0.441, 0.049)	0.116	− 0.212 (− 0.455, 0.022)	0.075
Currently working* (*n* = 135), number (%)	− 1.413 (− 2.384, − 0.442)	0.005	0.035 (− 0.853, 0.924)	0.937	− 1.380 (− 2.255, − 0.506)	0.002	− 0.031 (− 0.806, 0.745)	0.938	− 0.233 (− 0.433, − 0.033)	0.022	− 0.108 (− 0.322, 0.105)	0.318
Musculoskeletal disease measures												
PsA disease duration, years, mean (SD)	0.021 (− 0.052, 0.095)	0.563	–	–	− 0.012 (− 0.078, 0.054)	0.716	–	–	0.000 (− 0.015, 0.015)	0.983	–	–
CRP, mg/L, mean (SD)	− 0.020 (− 0.080, 0.039)	0.499	–	–	− 0.007 (− 0.061, 0.046)	0.785	–	–	− 0.005 (− 0.017, 0.008)	0.456	–	–
TJC68 (*n* = 138), mean (SD)	0.074 (0.031, 0.117)	0.001	− 0.020 (− 0.067, 0.028)	0.409	0.059 (0.020, 0.098)	0.004	0.004 (− 0.038, 0.046)	0.852	0.009 (0.000, 0.018)	0.056	0.005 (− 0.006, 0.016)	0.373
SJC66 (*n* = 138), mean (SD)	0.099 (− 0.375, 0.572)	0.680	–	–	− 0.009 (− 0.434, 0.417)	0.967	–	–	0.000 (− 0.096, 0.096)	0.996	–	–
MASES, range 0–13, mean (SD)	0.301 (0.152, 0.450)	0.000	0.068 (− 0.092, 0.229)	0.400	0.294 (0.162, 0.427)	0.000	0.052 (− 0.089, 0.193)	0.466	0.030 (− 0.002, 0,061)	0.065	− 0.013 (− 0.052, 0.026)	0.504
PROs												
Pain, VAS 0–100 mm, mean (SD)	0.078 (0.061, 0.095)	0.000	0.031 (0.006, 0.056)	0.015	0.071 (0.056, 0.086)	0.000	0.025 (0.003, 0.047)	0.024	0.006 (0.002, 0.010)	0.005	− 0.006 (− 0.012, 0.001)	0.087
Fatigue, NRS 0–10, mean (SD)	0.704 (0.557, 0.851)	0.000	0.434 (0.230, 0.638)	0.000	NA	NA	NA	NA	0.101 (0.067, 0.135)	0.000	0.106 (0.054, 0.157)	< 0.001
Sleep disturbance^†^, NRS 0–10, mean (SD)	NA	NA	NA	NA	0.568 (0.449, 0.686)	0.000	0.339 (0.183, 0.495)	< 0.001	0.062 (0.030, 0.095)	0.000	0.007 (− 0.040, 0.055)	0.759
Depression, range 1–3, mean (SD)	1.522 (0.722, 2.322)	0.000	− 1.180 (− 0.902, 0.542)	0.622	1.992 (1.317, 2.667)	0.000	1.242 (0.655, 1.830)	< 0.001	NA	NA	NA	NA
MHAQ, range 0–3, mean (SD)	4.153 (3.117, 5.188)	0.000	1.753 (0.225, 3.281)	0.025	3.331 (2.359, 4.304)	0.000	0.085 (− 1.287, 1.457)	0.902	0.392 (0.147, 0.637)	0.002	0.177 (− 0.171, 0.525)	0.316
Exercise ≥ 1 time per week, number (%)	− 1.060 (− 2.039, − 0.080)	0.034	− 0.710 (− 1.481, 0.060)	0.070	− 0.523 (− 1.413, 0.366)	0.247	–	–	− 0.109 (− 0.310, 0.092)	0.285	–	–
Comorbidity												
Comorbidities (*n* = 136), (range 0–8) mean (SD)	0.367 (− 0.164, 0.897)	0.174	− 0.074 (− 0.557, 0.410)	0.763	0.436 (− 0.038, 0.910)	0.071	0.001 (− 0.424, 0.427)	0.995	0.049 (− 0.059, 0.157)	0.369	–	–
Skin												
PASI, range 0–72 (*n* = 136), mean (SD)	0.106 (− 0.029, 0.241)	0.121	0.044 (− 0.074, 0.161)	0.463	0.060 (− 0.062, 0.182)	0.331	–	–	− 0.013 (− 0.040, 0.015)	0.354	–	–
Skin itching, (*n* = 121)	1.253 (0.040, 2.467)	0.043	− 0.858 (− 1.893, 0.177)	0.103	1.905 (0.860, 2.950)	0.000	0.776 (− 0.094, 1.647)	0.080	0.045 (− 0.201, 0.291)	0.719	–	–
Treatment												
Current bDMARD, number (%) (*n* = 136)	0.255 (− 0.806, 1.316)	0.635	–		0.366 (− 0.583, 1.315)	0.447	–	–	0.109 (− 1.106, 0.323)	0.318	–	–
Current csDMARD, number (%)	− 0.101 (− 1.107, 0.904)	0.842	–		0.415 (− 0.485, 1.316)	0.363	–	–	− 0.044 (− 0.247, 0.160)	0.672	–	–

Only variables with a *p* value < 0.20 in the univariate analysis were tested in the multivariate analysis, which was adjusted for age, gender, and BMI independent of their significance in the univariate analyses. Apart from age, gender, and BMI, only variables with at *p* value < 0.2 are displayed in the multivariate analysis

*NRS* numbering rating scale, *CI* confidence interval, *SD* standard deviation, *BMI* body mass index, *PsA* psoriatic arthritis, *CRP* C-reactive protein, *TJC* tender joint count, *SJC* swollen joint count, *MASES* Maastricht Ankylosing Spondylitis Enthesitis Score, *VAS* visual analogue scale, *MHAQ* Modified Health Assessment Questionnaire, *PASI* Psoriasis Area Severity Index, *bDMARD* biologic disease-modifying anti-rheumatic drug, *csDMARD* conventional synthetic DMARD

In the adjusted analysis as shown in Table 2, pain, fatigue, and higher MHAQ were independently associated with sleep disturbances. When fatigue was not in the model, pain and MHAQ were still associated with more sleep disturbances. Further, pain, sleep disturbances, and anxiety/depression were independently associated with fatigue whereas only fatigue was independently associated with anxiety/depression and anxiety. When the model also was adjusted with the interaction terms for pain, sleep disturbances, and fatigue (*p* = 0.898), fatigue still remained the only variable significantly associated (*p* < 0.001) with anxiety/depression. When TJC68 and MASES score was not in the adjusted models, the results remained the same. The same associations with sleep disturbances, fatigue, and anxiety/depression were found in the adjusted analysis with forward procedure.

## Discussion

The main finding in our study was that increased pain, fatigue, and impaired physical function were independently associated with increased sleep disturbances; increased pain, sleep disturbances, and anxiety/depression were independently associated with fatigue. Only increased fatigue was independently associated with anxiety/depression. For other measures including demographics and activity measures reflecting the inflammatory skin and the musculoskeletal disease process, none of these variables was independently associated with sleep disturbances, fatigue, and anxiety/depression.

In the literature, sleep disturbances have been reported to be of clinical relevance both in PsO [14–17] and PsA patients [18]. Sleep impairment has been reported to be more severe in PsA patients than in PsO patients [19, 20]. In a Nordic survey, sleep disturbances were reported by 16% of patients with PsO but by 45% of patients with PsA [21]. In our study, the prevalence of PsA patients reporting sleep disturbances was 38% (defined by ≥ 5 on a 0–10 NRS).

In randomized controlled trials (RCT), bDMARD treatment has been shown to improve sleep disturbance in PsO patients, highlighting its clinical significance [17, 22]. However, as has been reported and in this present study, musculoskeletal inflammation seems to have a more significant impact than skin involvement on sleep disturbances in PsA patients than skin involvement [18–20, 23]. In a telephone and e-mail survey of PsO patients, those reporting arthritis were found to be at highest risk of sleep disturbance [19]. However, they also found skin itching, pain of lesion, and impact on emotional well-being but not body surface covered with PsO, BMI, and therapy to be predictors of sleep interference. In our study, skin itching was in the univariate analysis, but not in the adjusted analysis, found to be associated with sleep disturbances. The lack of an association between skin involvement and sleep disturbances in our study may be explained by the relatively low activity of skin PsO, as reflected in the PASI scores.

Pain was in our study independently associated with sleep disturbances. We should keep in mind that our study is a cross-sectional study; thus, we are only exploring associations and not inferring causality between variables. However, most likely, pain is the driving force causing sleep disturbances in PsA patients and not the opposite. This view is supported by a recent study reporting increased prevalence of pain to be present even before the PsA diagnosis whereas the prevalence of sleep disturbances and fatigue first after the PsA diagnosis was increased compared with the background population [24]. However, there is data also supporting a reciprocal relationship with sleep and pain, where repeated nights of poor sleep worsen pain [25]. In our study, several independent variables for their associations with the dependent variables, e.g., sleep disturbances and fatigue, were studied which may reflect the same domain pain, e.g., global pain, TJC68, and MASES score. In the univariate analysis as shown in Table 2, both TJC68 and MASES score and pain were strongly associated with both sleep disturbances and fatigue; however, in the adjusted analysis, only pain remained statistically significantly associated. This is explained by a stronger association for pain than for TJC68 and MASES score with the dependent variables shown by higher *B* value for *Z* scores for pain compared with TJC68 and MASES score.

Not only does obesity increase the risk of developing PsO and PsA [26, 27] but also it increases the risk of other comorbidities which also may cause sleep problems and fatigue, including metabolic syndrome, hypertension, and diabetes [28, 29]. For BMI in our PsA patients, we found in univariate but not in the adjusted analysis a significant association between increased BMI and sleep disturbances, and a borderline significant association between BMI and fatigue. Interestingly, obesity has been reported to be the most important risk factor for obstructive sleep apnea which may cause fatigue [30].

Several other mechanisms than sleep disturbances may however also be involved causing fatigue in PsO and PsA patients. In PsA, the central nervous system is impacted either directly or indirectly by the inflammatory disease process; this may contribute to fatigue in inflammatory disorders including PsO and PsA [31]. Treatment with bDMARDs that effectively suppress inflammation has been shown to reduce fatigue both in PsO [32] and PsA patients [33]. Non-pharmacological interventions such as exercise may also improve sleep disturbances and fatigue in patients with inflammatory rheumatic disorders [34]. In a randomized controlled trial, high-intensity interval training in PsA patients was shown to improve fatigue whereas no negative effects were seen on measures reflecting disease activity or pain [35]. In our study, exercising was found to be associated with better sleep but not fatigue and this is only in the univariate analysis.

In patients with inflammatory disorders, including PsO and PsA, fatigue may have a significant impact on patients’ HRQoL. From the PsA patient perspective, fatigue is rated among the most important outcomes together with for example work, independence, physical function, and pain [9]. The burden and presence of fatigue in PsO and PsA patients has been documented in the literature [36–39]. In a cross-sectional study defining fatigue by the fatigue VAS (0–100) scale ≥ 50 and fatigue severity scale (FSS) ≥ 4, the prevalence of fatigue in PsO compared with healthy individuals was 51% and 4% using the VAS scale and 52% and 4% using the FSS scale [36]. In our study of PsA patients, the percentage of patients reporting fatigue was 44.5% (defined by ≥ 5 on a 0–10 NRS). In a Canadian study defining moderate to severe fatigue if FSS scores ≥ 5 and severe fatigue if FSS scores ≥ 7, the percentage of PsA patients with at least moderate fatigue was 49.5% and severe fatigue 28.7% [39].

In PsA patients, fatigue has been reported to be mostly related to impaired physical function, pain, and psychological stress [40]. However, in a recently published study using data from the Danish DANBIO registry, they found clinical inflammatory factors, along with disease duration and chronic pain, to be associated with fatigue [41]. One reason for these apparent differences with our study may be explained by that in the Danish study they used a principal component analysis whereas in our study the statistical analysis was based on the assessment of single variables using univariate and adjusted analyses. In our study, sleep disturbances, pain, and anxiety/depression were found to be independently associated with fatigue. The impact of pain and mental health on fatigue has been found by others [39, 42]. Different associations with fatigue have been identified in PsA patients, dependent on which disease outcome measures have been used. In a cross-sectional study of 246 PsA patients from 13 countries with 93.5% of the patients with current PsO covering more than 5% of the body surface, female gender, education level, tender joints, enthesitis, and PsO were found to be independently associated with fatigue in the adjusted analysis [38]. In our study, as also shown by others, skin involvement assessed by PASI score was not found to have a negative impact on fatigue in either univariate or adjusted analyses [39]. In both a cross-sectional and a longitudinal study of Canadian PsA patients, patient-reported measures of physical disability, pain, and psychological distress were most closely related to more fatigue [39, 42]. As also stated in the study by Husted et al., we also identified in the univariate analysis a large number of both demographic and disease measures to be significantly associated with fatigue in PsA as shown in Table 2; however, in the context of sleep disturbances, pain, and anxiety/depression in our PsA patient, they did not achieve statistical significance [39].

Depression and anxiety are among the comorbidities noted to be increased in PsA patients [29]. In our study, 33% of the PsA patients reported to be moderately anxious or depressed whereas only 5% reported to be extremely anxious or depressed. In a Nordic survey, 16.2% of the PsO and 34.9% of the PsA patients reported feelings of anxiety and depression [21]. In a recently published systematic review article of mental health in PsA including 24 studies, the authors concluded that anxiety and depression are highly prevalent among PsA patients [43]. However, large differences among the examined studies were reported for prevalences of depression in PsA, ranging from 5 to 51%. In another recently published systematic review article of depression and anxiety in PsA, where only 3 studies met the applied strict quality criteria for prevalence studies, the authors concluded that there is a moderate point prevalence of both depression and anxiety in PsA patients, which the authors emphasize is similar or slightly higher than reported in the general population and comparable to that seen in other rheumatic diseases [44]. This view that anxiety and depression are a less significant comorbidity in PsA has also recently been supported by two studies not included in the two review articles. In the study by Michelsen et al., HRQoL both for the SF-36 mental and the physical sum scores were significantly lower in PsA patients than in the background population [3]. However, the difference between PsA patients and controls was less for the mental (47.1 vs 50.0) than for the physical sum score (30.5 vs 50.0). Further, in a population-based survey study, anxiety and depression were not found to be more present in PsA patients than in the background population [24]. The lack of an association between skin involvement and anxiety/depression in our PsA patients is most likely explained by the low PASI score of only 2.6.

The low number of patients reporting more severe anxiety/depression in our study may be biased by the question we used with only three response alternatives. Thus, the lack of consistency reported in the literature may be explained by study design, questionnaires used, and the heterogeneity of study populations examined in the various studies.

In our study, smoking, not being employed/working, impaired physical function, pain, sleep disturbances, and fatigue were in the univariate analysis associated with increased anxiety/depression, but only fatigue was found to be independently associated with increased anxiety/depression. The interaction however between pain, sleep disturbances, and fatigue may all contribute to increased risk of anxiety/depression in PsA.

Increased disease activity using objective measures has to our knowledge not been identified as a risk factor causing anxiety and/or depression in PsA. In a study by Michelsen et al., anxiety/depression was shown to reduce the likelihood of remission using various composite scores, e.g., DAS28 and DAPSA [45]. This however was explained by the subjectively weighted measures in the composite scores (patient global assessment and tender joint count) but not by acute phase reactants and swollen joints, the objectively weighted measures in the composite scores [45]. In a recently published study, fibromyalgia was also reported to be the most important factor reducing the possibility to achieve low or minimal disease activity in PsA [46]. However, in contrast to this, treatment with bDMARDs has been shown to reduce depressive symptoms and improve mental status in both PsO [47] and PsA patients [4, 48, 49].

Limitations of our study include study design (cross sectional) only allowing to explore for associations and not causality and the lack of an age- and gender-matched control group. A strength of our study is the broad specter of variables used to explore for associations with the dependent variables. However, this may also be seen as a limitation of the study including many independent variables in the adjusted analysis taking into account that a rather small number of patients was included. The same results however were obtained when the adjusted analysis was performed with forward procedure in the multivariate linear regression analysis as performed with enter procedure. Other limitations of this study have also been addressed above. The internal validity is judged to be satisfactory as we have previously shown that the examined cohort of PsA patients was found to be fairly representative for the whole PsA outpatient clinic cohort [11].

## Conclusions

The prevalence of sleep disturbances (38.0%), fatigue (44.5%), and anxiety/depression (38.0%) in our PsA patients seems to be more in the middle to high end of those reported in the literature. No skin measures, neither PASI score nor skin itching, was found to be associated with sleep disturbances or fatigue. Only musculoskeletal involvement in PsA, e.g., expressed as pain, was independently associated with increased sleep disturbances and fatigue. Only fatigue and no measures reflecting the inflammatory skin or musculoskeletal disease process was found to be associated with anxiety/depression. Our data highlights the need for clinicians to be aware of the presence and importance of these domains, judged by patients to be of particular importance, and take them into account when treating PsA patients because focusing only on objective measures reflecting disease activity in the follow-up of PsA patients would give a false impression of the burden of the disease for the PsA patients.

## Data Availability

The datasets used and/or analyzed during the current study are available from the corresponding author on reasonable request.

## Abbreviations

PsA: Psoriatic arthritis
HRQoL: Health-related quality of life
PsO: Psoriasis
CASPAR: Classification of Psoriatic Arthritis criteria
NRS: Numeric rating scale
BMI: Body mass index
CRP: C-reactive protein
TJC: Tender joint count
SJC: Swollen joint count
MASES: Maastricht Ankylosing Spondylitis Enthesitis Score
mHAQ: Modified Health Assessment Questionnaire
CVD: Cardiovascular disease
PASI: Psoriasis Area Severity Index
DMARDs: Disease-modifying anti-rheumatic drugs
SD: Standard deviation
